# One-Pot Synthesis of Dihydrothiazole-Hydrazone Hybrids and Evaluation of Their Absorption and Emission Properties

**DOI:** 10.1007/s10895-026-04955-6

**Published:** 2026-09-29

**Authors:** Charan D. C., Yogeesh M., Nitinkumar S. Shetty

**Affiliations:** Manipal Institute of Technology, Manipal Academy of Higher Education, Manipal, India

**Keywords:** Dihydrothiazole-hydrazone hybrids, Schiff base, One-pot synthesis, Cyclization, Substituent effects

## Abstract

A simple, one-pot method for the synthesis of seven novel dihydrothiazole-hydrazone compounds is presented, employing a condensation-cyclization protocol, with the products characterized by FTIR, ^1^H NMR, ^13^C NMR, and ESI-MS. Thiazoles and hydrazones are distinguished by versatile chelating properties and fluorescent conjugated scaffolds, and their combination can be considered a promising route to obtaining tunable fluorophores. According to the UV-Vis absorption and fluorescence data, all seven compounds demonstrated absorption maxima at 412–420 nm and emitted a yellow-green light at 554–575 nm. The substituent present at the C-4 position of the dihydrothiazole ring governed the emission wavelength and intensity. Electron-donating groups increased emission intensity and shifted the emission to longer wavelengths, whereas electron-withdrawing groups quenched fluorescence, likely via a photoinduced electron transfer (PET) deactivation mechanism. The Stokes shifts (142–155 nm) also followed the same substituent-dependent pattern. Moderate fluorescence quantum yields (1.64–5.54%) were observed for the thiazoles, indicating that the presence of different functional groups influenced the efficiency of excited-state relaxation. The way substitution influences absorption and emission behavior establishes the dihydrothiazole-hydrazone core as a versatile unit that can be tuned to serve as a fluorophore for sensing, biological, and optoelectronic applications.

**Graphical Abstract:**

**Figure:**
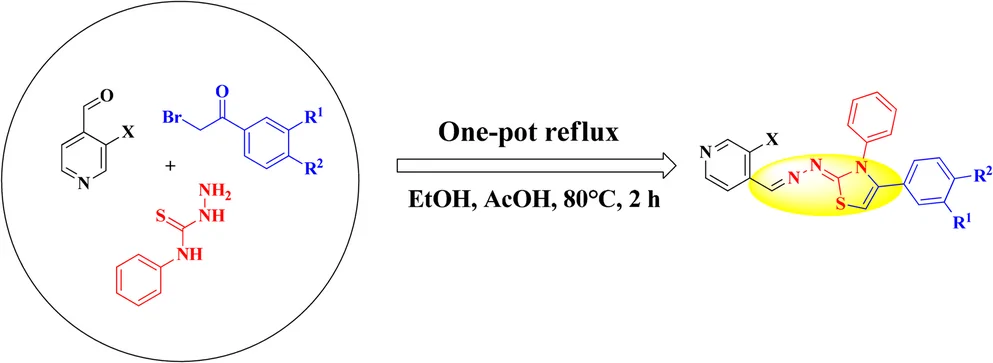

**Supplementary Information:**

The online version contains supplementary material available at https://doi.org/10.1007/s10895-026-04955-6.

## Introduction

Among heterocyclic compounds of importance, thiazoles occupy a prominent position and have found wide-ranging applications in medicinal chemistry and materials science [1, 2]. They contain a soft sulfur atom along with an imine nitrogen and have also been found to be effective chelating agents and participants in diverse biological systems [1, 3]. Upon modification of the thiazole core’s absorption and emission behavior by substituents, this has been proven useful for Zn^2+^ sensing through photoluminescence changes [4], solid-state emissive crystals for photonic and optical devices [5], solvatochromic fluorophores exhibiting solvatochromism in response to solvent polarity [6], dyes with nonlinear optical properties [7], and coplanar organic semiconducting materials [8]. Moreover, it has led to derivatives that exhibit improved biological effectiveness and selectivity [9, 10].

Similarly, among Schiff base derivatives, hydrazones stand out as a particularly important subclass. They are obtained by condensing hydrazine derivatives with aldehydes or ketones and exhibit antitubercular [11] and antioxidant activity [12]. Moreover, hydrazones serve as efficient chelating agents [13, 14]. Their coordinating ability, combined with the rigidity of the C = N bond, significantly increases the stability of the resulting metal complexes [14]. This same rigidity restricts molecular rotation, resulting in a more planar system that increases interactions with biological targets, thereby making them essential linkers in hybrid molecular design [15]. Fluorescence is also affected by this rigidity, as a major non-radiative decay route is blocked; this same principle was exploited in Schiff base-derived fluorescent molecular rotors that rely on C = N bond rotation, such as viscosity sensors [16]. In the design of chemosensors for various analytes, hydrazones have been employed in fluorescence- and colorimetry-based approaches [17–19], and they also serve as fluorescent materials and molecular switches that respond to external stimuli [20].

As seen earlier, both thiazoles and hydrazones are extremely useful, and it makes sense that their combination would broaden their functional scope. This synergy gives rise to thiazole-hydrazone hybrids, a key bis-heterocyclic scaffold in drug discovery that delivers superior biological efficacy and greater versatility compared to either component alone [21]. An example of this is evident in the development of a series of thiazole-hydrazone hybrids targeting Akt signaling in non-small-cell lung cancer [22]. Similarly, furan-thiazole-hydrazone derivatives have demonstrated antitubercular activity comparable to that of the standard drug pyrazinamide [23]. This class of hybrids has also found application in fluorescence-based sensing, where a benzothiazole-hydrazone probe enabled the selective detection of Ga^3+^ and pyrophosphate ions [24]. Even in the field of catalysis, a molybdenum (VI) complex derived from a thiazole-hydrazone ligand has been identified as an efficient homogeneous catalyst for olefin epoxidation [25]. As seen above, one can reasonably conclude that combining these two units into a single framework might result in enhanced characteristics. These hybrids are commonly synthesized via a Hantzsch-type approach, which involves a cyclocondensation step in which an α-haloketone reacts with a thiosemicarbazide or thiourea-hydrazide, simultaneously forming the thiazole ring and installing the hydrazone linkage in a single operation [26]. Recent trends in green chemistry have shifted synthesis procedures towards one-pot methods assisted by microwave irradiation, as these are known to reduce reaction times and improve yields compared with conventional techniques [27]. Therefore, a one-pot method provides a straightforward approach to obtaining thiazole-hydrazone compounds without the need for separate ring-formation and condensation steps or the isolation of intermediates.

Our idea was that an aromatic aldehyde bearing a free NH_2_ group could react to form a suitable intermediate, which could then undergo cyclization with α-bromoacetophenone, thereby affording compounds containing both the thiazole and hydrazone units. Upon reviewing the literature, we found that Palabindela and coworkers [28] had employed a similar strategy, condensing an aromatic aldehyde with 4-phenyl-3-thiosemicarbazide to form an arylidene-N-phenylhydrazinecarbothioamide intermediate, which was then cyclized using various α-bromoacetophenones to yield a series of N-phenyl-substituted thiazole-hydrazone hybrids. This two-step condensation-cyclization sequence offers a straightforward and effective route to a wide range of dihydrothiazole-hydrazone compounds, requiring neither specialized reagents nor harsh reaction conditions. In the present study, a similar synthetic approach was adopted, in which an aldehyde was condensed with 4-phenyl-3-thiosemicarbazide to form the corresponding thiosemicarbazone, which was then cyclized with appropriate α-bromoacetophenones to construct the dihydrothiazole-hydrazone scaffold. The synthesized compounds were characterized using FTIR, ^1^H and ^13^C NMR, and mass spectrometry. Subsequently, UV-Vis and photoluminescence spectroscopy were used to investigate their absorption and emission behavior and to evaluate the influence of substituent electronics on these properties.

## Experimental

### Materials and Instrumentation

All reagents used in the synthesis and analysis were of commercial grade and used directly without further purification. The purity of the synthesized compounds was assessed by thin-layer chromatography (TLC), and melting points were determined in open capillary tubes using a Thiele tube apparatus. Infrared spectra were acquired in the solid state using a Shimadzu QATR-S IR Spirit spectrophotometer employing the attenuated total reflectance (ATR) technique over a spectral range of 400–4000 cm^− 1^ at a resolution of 4 cm^− 1^ with 40 scans, at room temperature. A Bruker spectrometer was used to record ^1^H NMR (400 MHz) and ^13^C NMR (100 MHz) spectra, with DMSO-d_6_ as the deuterated solvent and tetramethylsilane (TMS) as the internal standard. UV-Vis spectral absorptions were acquired in DMF at a concentration of 1 × 10^− 4^ M using a 1 cm quartz cuvette, with DMF as the reference blank, on a Shimadzu UV-1900i spectrophotometer over the range of 350–600 nm. Photoluminescence spectra were recorded at room temperature in solution state using a JASCO FP-8500 spectrofluorometer equipped with a 450 W xenon lamp, at a concentration of 1 × 10^− 4^ M. The quantum efficiency of 5a-g was measured by the absolute method in solution at a concentration of 1 × 10^− 4^ M in DMF, using a Horiba FluoroMax Plus spectrofluorometer equipped with an integrating sphere coated with Spectralon. No standard reference was required for an absolute method of determination. The instrument’s quantum yield calculation software was used to determine the quantum yield at an excitation wavelength of 370 nm, based on the ratio of emitted photons to absorbed photons:

$$\phi_f\;=\;\frac{\int\;I_{em}\left(\lambda\right)d\lambda-\;\int\;I_{ref\;2}\left(\lambda\right)d\lambda}{\;\int\;I_{ref\;1}\left(\lambda\right)d\lambda-\;\int\;I_{ex}\left(\lambda\right)d\lambda}$$

Where *I*_*ref 1*_*(λ)* and *I*_*ex*_*(λ)* are the excitation light profiles of the blank solvent and the sample, respectively, and *I*_*ref 2*_*(λ)* and *I*_*em*_*(λ)* are the emission spectra of the solvent and the sample, respectively.

### General Procedure for the Synthesis of Compounds 5a–g

**Scheme 1:**
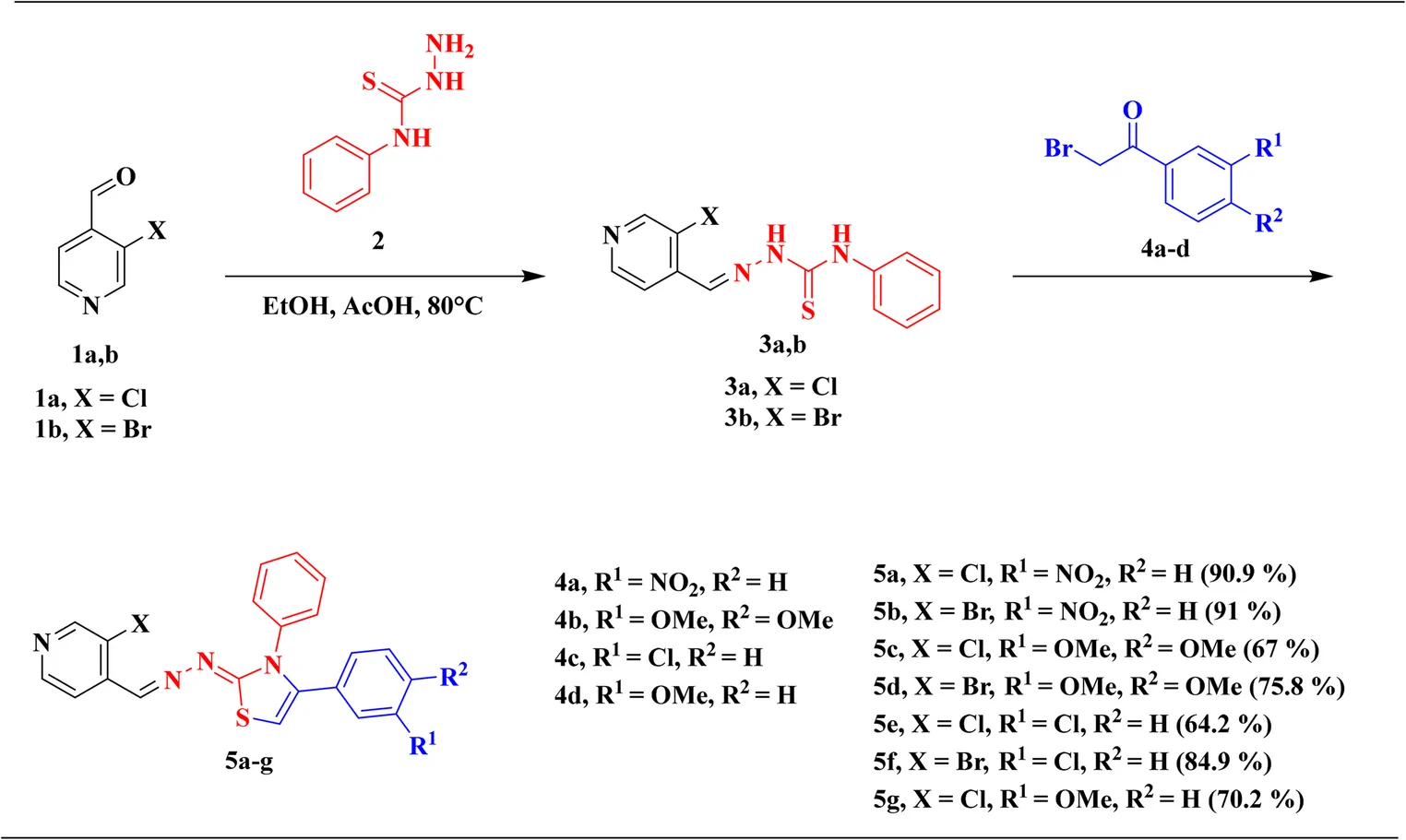
General route for the synthesis of compounds 5a–g

**Fig. 1:**
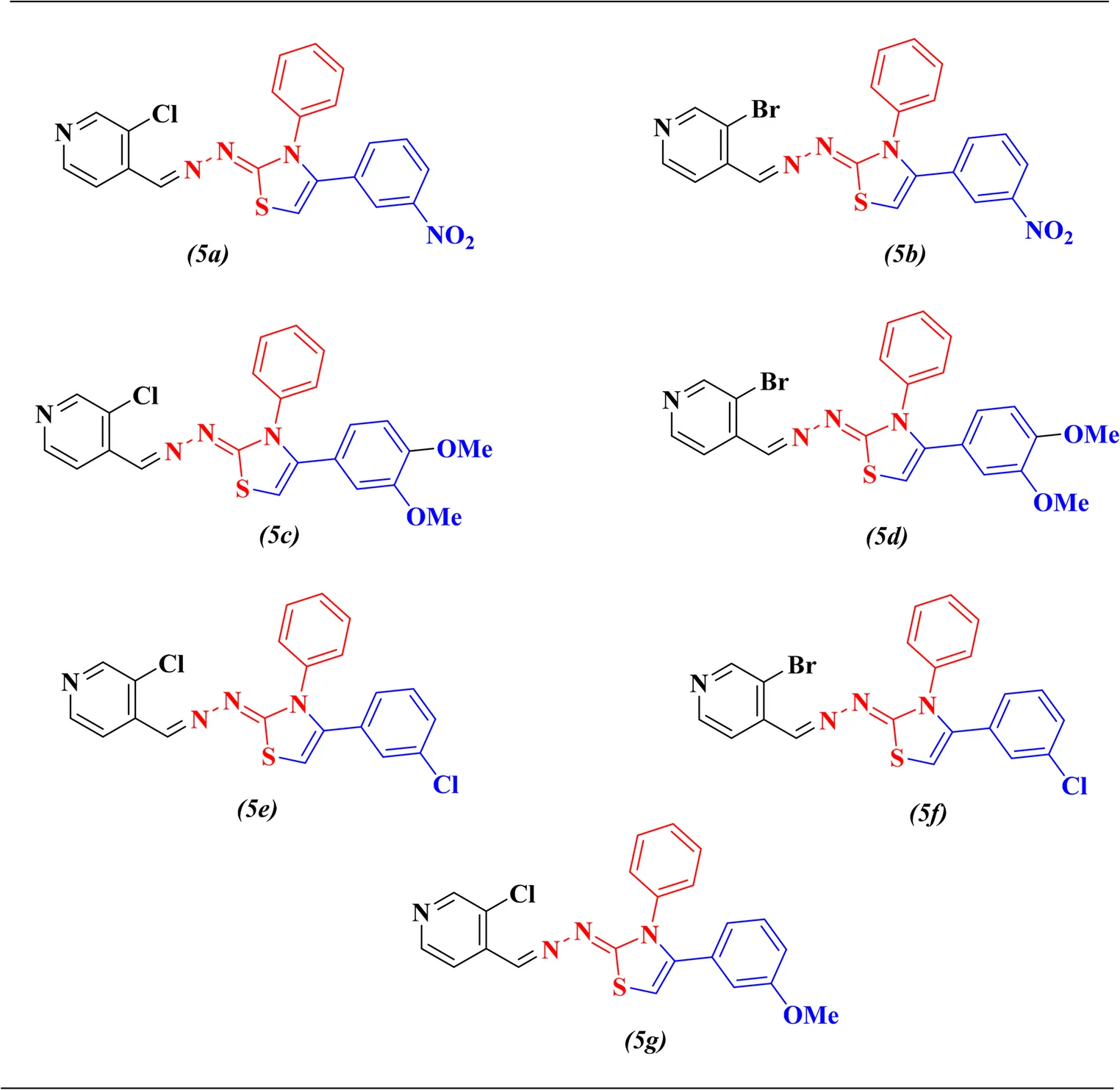
Structures of synthesized dihydrothiazole-hydrazone derivatives 5a–g

A mixture of 3-haloisonicotinaldehyde (1 equiv.) (1) and 4-phenyl-3-thiosemicarbazide (1 equiv.) (2) in ethanol (1–2 mL) was treated with glacial acetic acid (2 drops), and the mixture was refluxed at 80 °C. The progress of the reaction was monitored by TLC, which indicated complete consumption of the starting aldehyde after 1 h. The corresponding 2-bromo-1-(substituted phenyl)ethan-1-one (1 equiv.) (4) was then added to the in situ formed thiosemicarbazone intermediate (3), and heating under reflux at 80 °C was continued for an additional hour, with reaction completion again confirmed by TLC. The resulting precipitate was collected by filtration while the mixture was still hot, and the solid was washed with ethanol. The crude product was dried in an oven at 70 °C and subsequently recrystallized from a methanol/THF mixture to afford the desired products 5a–g (Scheme 1). The structures of the synthesized dihydrothiazole-hydrazone derivatives 5a–g are shown in Fig. 1.

### Spectral Characterization

#### Synthesis of 2-((Z)-((3-chloropyridin-4-yl)methylene)hydrazono)-4-(3-nitrophenyl)-3-phenyl-2,3-dihydrothiazole (5a)

Brown solid; yield 90.9%; m. p 200–202 °C; FTIR (ATR, ν cm^− 1^): 3064 cm^− 1^ (Ar, CH, str.),

1622.82 cm^− 1^ (C = N, str.), 1585.74 cm^− 1^ (Ar, C = C, str.), 1525 cm^− 1^ (NO_2_, asym. str.), 1353 cm^− 1^ (NO_2_, sym. str.), 1193 cm^− 1^ (Ar, C–C, str.) (Fig. S1); ^1^H NMR (DMSO-d_6_, 400 MHz) d, ppm (J, Hz): 8.655 (s, 1H, ArH), 8.540 (d, J = 4.8, 1H, ArH), 8.277 (s, 1H, HC = N), 8.126 (d, J = 8, 1H, ArH), 8.022 (s, 1H, ArH), 7.865 (d, J = 5.2, 1H, ArH), 7.625 (d, J = 8, 1H, ArH), 7.551 (t, J = 8, 1H, ArH), 7.364–7.444 (m, 5 H, ArH), 7.100 (s, 1H, ArH) (Fig. S2); ^13^C NMR (DMSO-d_6_, 100 MHz) d, ppm: 174.72, 147.81, 147.10, 144.54, 143.51, 143.25, 138.59, 136.80, 135.47, 131.85, 130.40, 130.25, 129.80, 129.42, 129.15, 123.98, 123.78, 121.36, 106.82 (Fig. S3); Molecular formula: C_21_H_14_ClN_5_O_2_S; ESI-MS (m/z) = 436.37 [M + H]^+^, 438.38 [(M + 2) + H]^+^ (Fig. S4).

#### Synthesis of 2-((Z)-((3-bromopyridin-4-yl)methylene)hydrazono)-4-(3-nitrophenyl)-3-phenyl-2,3-dihydrothiazole (5b)

Orange solid; yield 91%; m. p 202–205 °C; FTIR (ATR, ν cm^− 1^): 3063.10 cm^− 1^ (Ar, CH, str.), 1622.82 cm^− 1^ (C = N, str.), 1587.17 cm^− 1^ (Ar, C = C, str.), 1525.85 cm^− 1^ (NO_2_, asym. str.), 1350.45 cm^− 1^ (NO_2_, sym. str.), 1192.16 cm^− 1^ (Ar, C–C, str.) (Fig. S5); ^1^H NMR (DMSO-d_6_, 400 MHz) d, ppm (J, Hz): 8.764 (s, 1H, ArH), 8.571 (d, J = 4.8, 1H, ArH), 8.214 (s, 1H, HC = N), 8.124 (d, J = 8, 1H, ArH), 8.024 (s, 1H, ArH), 7.858 (d, J = 5.2, 1H, ArH), 7.639 (d, J = 7.6, 1H, ArH), 7.551 (t, J = 8, 1H, ArH), 7.349–7.447 (m, 5 H, ArH), 7.109 (s, 1H, ArH) (Fig. S6); ^13^C NMR (DMSO-d_6_, 100 MHz) d, ppm: 174.67, 149.54, 147.82, 145.55, 144.98, 144.88, 138.58, 136.81, 135.46, 131.87, 130.39, 129.80, 129.41, 129.15, 123.98, 123.78, 121.72, 120.60, 106.76 (Fig. S7); Molecular formula: C_21_H_14_BrN_5_O_2_S; ESI-MS (m/z) = 480.33 [M + H]^+^, 482.35 [(M + 2) + H]^+^ (Fig. S8).

#### Synthesis of 2-((Z)-((3-chloropyridin-4-yl)methylene)hydrazono)-4-(3,4- dimethoxyphenyl)-3-phenyl-2,3-dihydrothiazole (5c)

Pale yellow solid; yield 67%; m. p 178–180 °C; FTIR (ATR, ν cm^− 1^): 3080.21 cm^− 1^ (Ar, CH, str.), 2909.09 cm^− 1^ (OMe, C–H, asym. str.), 2830.66 cm^− 1^ (OMe, C–H, sym. str.), 1622.82 cm^− 1^ (C = N, str.), 1590.02 cm^− 1^ (Ar, C = C, str.), 1270.59 cm^− 1^ (C–O–C, str.) (Fig. S9); ^1^H NMR (DMSO-d_6_, 400 MHz) d, ppm (J, Hz): 8.639 (s, 1H, ArH), 8.523 (d, J = 4.8, 1H, ArH), 8.240 (s, 1H, HC = N), 7.853 (d, J = 4.8, 1H, ArH), 7.320–7.442 (m, 5 H, ArH), 6.805–6.861 (m, 2 H, ArH), 6.761 (s, 1H, ArH), 6.614 (s, 1H, ArH), 3.699 (s, 3 H, OCH_3_), 3.475 (s, 3 H, OCH_3_) (Fig. S10); ^13^C NMR (DMSO-d_6_, 100 MHz) d, ppm: 174.76, 149.55, 148.35, 147.60, 145.13, 143.00, 142.83, 141.06, 137.44, 130.05, 129.65, 129.23, 129.14, 122.61, 121.94, 112.09, 112.60, 111.74, 103.29, 55.88 (Fig. S11); Molecular formula: C_23_H_19_ClN_4_O_2_S; ESI-MS (m/z) = 451.41 [M + H]^+^, 453.42 [(M + 2) + H]^+^ (Fig. S12).

#### Synthesis of 2-((Z)-((3-bromopyridin-4-yl)methylene)hydrazono)-4-(3,4-dimethoxyphenyl)-3-phenyl-2,3-dihydrothiazole (5d)

Pale yellow solid; yield 75.8%; m. p 182–186 °C; FTIR (ATR, ν cm^− 1^): 3103.03 cm^− 1^ (Ar, CH, str.), 2967.56 cm^− 1^ (OMe, C–H, asym. str.), 2837.79 cm^− 1^ (OMe, C–H, sym. str.), 1622.82 cm^− 1^ (C = N, str.), 1595.72 cm^− 1^ (Ar, C = C, str.), 1260.61 cm^− 1^ (C–O–C, str.) (Fig. S13); ^1^H NMR (DMSO-d_6_, 400 MHz) d, ppm (J, Hz): 8.742 (s, 1H, ArH), 8.538 (d, J = 5.2, 1H, ArH), 8.177 (s, 1H, HC = N), 7.840 (d, J = 5.2, 1H, ArH), 7.321–7.446 (m, 5 H, ArH), 6.802–6.862 (m, 2 H, ArH), 6.763 (s, 1H, ArH), 6.616 (s, 1H, ArH), 3.699 (s, 3 H, OCH_3_), 3.476 (s, 3 H, OCH_3_) (Fig. S14); ^13^C NMR (DMSO-d_6_, 100 MHz) d, ppm: 174.81, 149.79, 149.55, 148.35, 145.25, 145.02, 144.64, 141.07, 137.43, 129.66, 129.22, 129.15, 122.60, 121.94, 121.52, 120.48, 112.60, 111.74, 103.32, 55.70 (Fig. S15); Molecular formula: C_23_H_19_BrN_4_O_2_S; ESI-MS (m/z) = 495.40 [M + H]^+^, 497.41 [(M + 2) + H]^+^ (Fig. S16).

#### Synthesis of 4-(3-chlorophenyl)-2-((Z)-((3-chloropyridin-4-yl)methylene)hydrazono)-3-phenyl-2,3-dihydrothiazole (5e)

Olive green solid; yield 64.2%; m. p 118–120 °C; FTIR (ATR, ν cm^− 1^): 3057.40 cm^− 1^ (Ar, CH, str.), 1622.82 cm^− 1^ (C = N, str.), 1591.44 cm^− 1^ (Ar, C = C, str.), 1189.30 cm^− 1^ (Ar, C–C, str.) (Fig. S17); ^1^H NMR (DMSO-d_6_, 400 MHz) d, ppm (J, Hz): 8.651 (s, 1H, ArH), 8.533 (d, J = 5.2, 1H, ArH), 8.258 (s, 1H, HC = N), 7.956 (d, J = 4.8, 1H, ArH), 7.248–7.443 (m, 8 H, ArH), 7.123 (d, J = 7.6, 1H, ArH), 6.953 (s, 1H, ArH) (Fig. S18); ^13^C NMR (DMSO-d_6_, 100 MHz) d, ppm: 174.59, 147.71, 145.25, 143.33, 142.85, 139.35, 137.03, 133.34, 132.40, 130.55, 130.13, 129.68, 129.27, 129.19, 129.12, 128.90, 127.77, 121.19, 105.58 (Fig. S19); Molecular formula: C_21_H_14_Cl_2_N_4_S; ESI-MS (m/z) = 425.36 [M + H]^+^, 427.36 [(M + 2) + H]^+^ (Fig. S20).

#### Synthesis of 2-((Z)-((3-bromopyridin-4-yl)methylene)hydrazono)-4-(3-chlorophenyl)-3-phenyl-2,3-dihydrothiazole (5f)

Reddish brown solid; yield 85.9%; m. p 140–142 °C; FTIR (ATR, ν cm^− 1^): 3057.40 cm^− 1^ (Ar, CH, str.), 1621.39 cm^− 1^ (C = N, str.), 1590.02 cm^− 1^ (Ar, C = C, str.), 1186.45 cm^− 1^ (Ar, C–C, str.) (Fig. S21); ^1^H NMR (DMSO-d_6_, 400 MHz) d, ppm (J, Hz): 8.750 (s, 1H, ArH), 8.546 (d, J = 4.8, 1H, ArH), 8.195 (s, 1H, HC = N), 7.840 (d, J = 5.2, 1H, ArH), 7.248–7.446 (m, 8 H, ArH), 7.123 (d, J = 8, 1H, ArH), 6.954 (s, 1H, ArH) (Fig. S22); ^13^C NMR (DMSO-d_6_, 100 MHz) d, ppm: 174.59, 150.02, 145.55, 144.35, 139.34, 137.18, 137.03, 133.34, 132.40, 130.55, 129.69, 129.27, 129.19, 129.12, 128.89, 127.77, 121.59, 120.58, 105.58 (Fig. S23); Molecular formula: C_21_H_14_BrClN_4_S; ESI-MS (m/z) = 469.34 [M + H]^+^, 471.35 [(M + 2) + H]^+^, 473.34 [(M + 4) + H]^+^ (Fig. S24).

#### Synthesis of 2-((Z)-((3-chloropyridin-4-yl)methylene)hydrazono)-4-(3-methoxyphenyl)-3-phenyl-2,3-dihydrothiazole (5 g)

Brown solid; yield 70.2%; m. p 150–152 °C; FTIR (ATR, ν cm^− 1^): 3054.55 cm^− 1^ (Ar, CH, str.), 2967.56 cm^− 1^ (OMe, C–H, asym. str.), 2849.20 cm^− 1^ (OMe, C–H, sym. str.), 1614.26 cm^− 1^ (C = N, str.), 1555.79 cm^− 1^ (Ar, C = C, str.), 1270.59 cm^− 1^ (C–O–C, str.) (Fig. S25); ^1^H NMR (DMSO-d_6_, 400 MHz) d, ppm (J, Hz): 8.644 (s, 1H, ArH), 8.528 (d, J = 5.2, 1H, ArH), 8.250 (s, 1H, HC = N), 7.855 (d, J = 5.2, 1H, ArH), 7.322–7.433 (m, 5 H, ArH), 7.163 (t, J = 8, 1H, ArH), 6.855 (s, 1H, ArH), 6.831 (d, J = 8.4, 1H, ArH), 6.778 (d, J = 7.6, 1H, ArH), 6.708 (s, 1H, ArH), 3.584 (s, 3 H, OCH_3_) (Fig. S26); ^13^C NMR (DMSO-d_6_, 100 MHz) d, ppm: 174.70, 159.18, 147.85, 145.42, 143.15, 142.70, 140.77, 137.33, 131.60, 130.07, 129.90, 129.63, 129.49, 129.11, 121.32, 121.08, 115.16, 114.41, 104.47, 55.47 (Fig. S27); Molecular formula: C_22_H_17_ClN_4_OS; ESI-MS (m/z) = 421.40 [M + H]^+^, 423.41 [(M + 2) + H]^+^ (Fig. S28).

## Results and Discussion

### Chemistry

In this study, we synthesized seven novel dihydrothiazole-hydrazone hybrid derivatives using the route outlined in Scheme 1. The synthesis involved the initial condensation of 3-haloisonicotinaldehydes with 4-phenyl-3-thiosemicarbazide to afford the corresponding thiosemicarbazone intermediates, which were subsequently cyclized with 2-bromo-1-(substituted phenyl)ethan-1-ones to furnish the target compounds 5a–g in good yields (64–91%). FTIR spectra of all derivatives exhibited a characteristic azomethine C = N stretch near 1614–1623 cm^− 1^, aromatic C = C stretching in the range 1555–1595 cm^− 1^, and aromatic C–H stretching near 3054–3103 cm^− 1^. Compounds 5a and 5b, bearing the 3-nitrophenyl substituent, additionally displayed asymmetric and symmetric NO_2_ stretching bands near 1525 and 1350 cm^− 1^, respectively, while the methoxy-substituted derivatives 5c, 5d, and 5 g showed characteristic OMe C–H stretches at 2830–2967 cm^− 1^ along with a C–O–C stretch near 1260–1270 cm^− 1^, confirming the presence of these functional groups.

The ^1^H NMR spectra of all derivatives displayed a distinct singlet near δ 8.177–8.277 ppm, assignable to the azomethine (HC = N) proton, confirming the formation of the hydrazone linkage. The singlet observed in the range of δ 6.614–7.109 ppm, which is indicative of the proton of the dihydrothiazole ring of all 5a-g. Followed by compounds 5c, 5d, and 5 g, which contain the OCH3 group at the C-4 position, appear as a singlet in NMR spectra. The dimethoxy-substituted derivatives showed two distinct OCH_3_ signals corresponding to the two non-equivalent methoxy groups: 5c at δ 3.475 and 3.699 ppm, and 5d at δ 3.476 and 3.699 ppm. The monomethoxy derivative 5 g, in contrast, displayed only a single OCH_3_ signal at δ 3.584 ppm. ^13^C NMR spectra corroborated these assignments, with a peak observed at δ 174.59–174.81 ppm assigned to the dihydrothiazole C-2 carbon, aromatic and pyridine carbons distributed across δ 103.29–159.18 ppm, and the methoxy carbons of 5c, 5d, and 5 g appearing near δ 55.47–55.88 ppm. ESI-MS spectra of all seven compounds showed [M + H]^+^ peaks accompanied by the expected [(M + 2) + H]^+^ isotope peaks, consistent with the presence of chlorine and/or bromine, confirming the proposed molecular formulae.

### Absorption and Emission Studies

#### UV-Vis Spectral Analysis

**Fig. 2:**
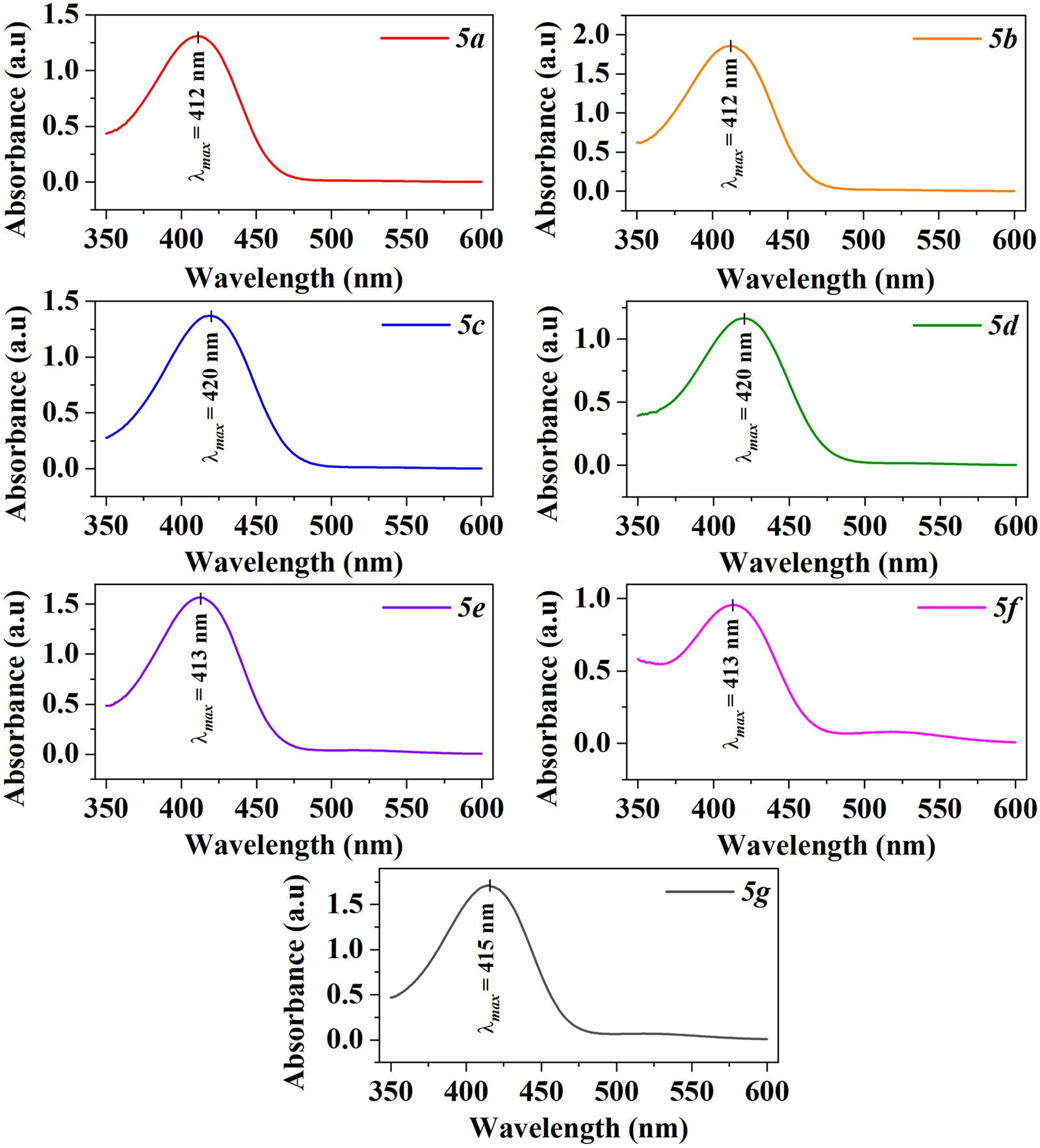
UV-Vis absorption spectra of compounds 5a–g in DMF (1 × 10^− 4^ M)

The UV-Vis absorption spectra of compounds 5a–g were obtained in N, N-dimethylformamide (DMF) at a concentration of 1 × 10^− 4^ M, at room temperature, over the wavelength range 350–600 nm (Fig. 2). The extended π-conjugation within the hydrazone-dihydrothiazole framework of the compounds allows absorption at longer wavelengths in the visible region of the electromagnetic spectrum [29]. All the compounds showed only one absorption peak in the range 412–420 nm, indicating that the major electronic transition was the π → π^*^ transition, with the possible presence of an n → π^*^ transition at the lower energy tail of the band [30].

The absorption maximum (λ_max_) was found to be dependent on the type of aryl substituent at the C-4 position of the dihydrothiazole scaffold [31]. Compounds 5c and 5d, bearing the electron-donating 3,4-dimethoxyphenyl substituent, demonstrated the largest bathochromic shift (λ_max_ = 420 nm) compared to the nitrophenyl analogues (5a, 5b). The observed redshift could be explained by the stronger electron-donating ability of the two methoxy groups, which extend conjugation via resonance interactions with the dihydrothiazole ring [32]. On the other.

hand, the presence of the highly electron-withdrawing 3-nitrophenyl group in 5a and 5b resulted in the greatest hypsochromic shift (λ_max_ = 412 nm) among all the studied structures. This blue shift is consistent with the electron-withdrawing nature of the nitro group, which leads to a higher energy gap in the π → π^*^ transition [33]. Furthermore, within each substitution pair, the replacement of chlorine by bromine at the C-3 position of the pyridine ring produced no appreciable shift in l_max_, suggesting that the halogen substituent at this position has a negligible influence on the π-conjugated system responsible for the observed electronic transitions.

Compounds 5e and 5f, bearing a 3-chlorophenyl substitution, exhibited an absorption maximum at 413 nm, which lies between those of compounds bearing methoxyphenyl and nitrophenyl substitutions. This result is due to the weak inductive electron-withdrawing effect of the chlorine atom, which leads to a slight hypsochromic shift compared with the electron-donating methoxy substituent [34]. The compound 5 g, with a 3-methoxyphenyl group, gave a λ_max_ of 415 nm, indicating that the bathochromic shift depends on the electron-donating ability and the number of alkoxy substituents in the aryl ring, as compared with compounds 5 g, 5c, and 5d [35]. Thus, it can be concluded that the order of bathochromic shift of l_max_ among different compounds of the series is: 5c and 5d (420 nm) > 5 g (415 nm) > 5e and 5f (413 nm) > 5a and 5b (412 nm), consistent with which is indicative of the electronic influence of the aryl substitution at the C-4 position in the dihydrothiazole ring.

#### Photoluminescence Analysis

**Fig. 3:**
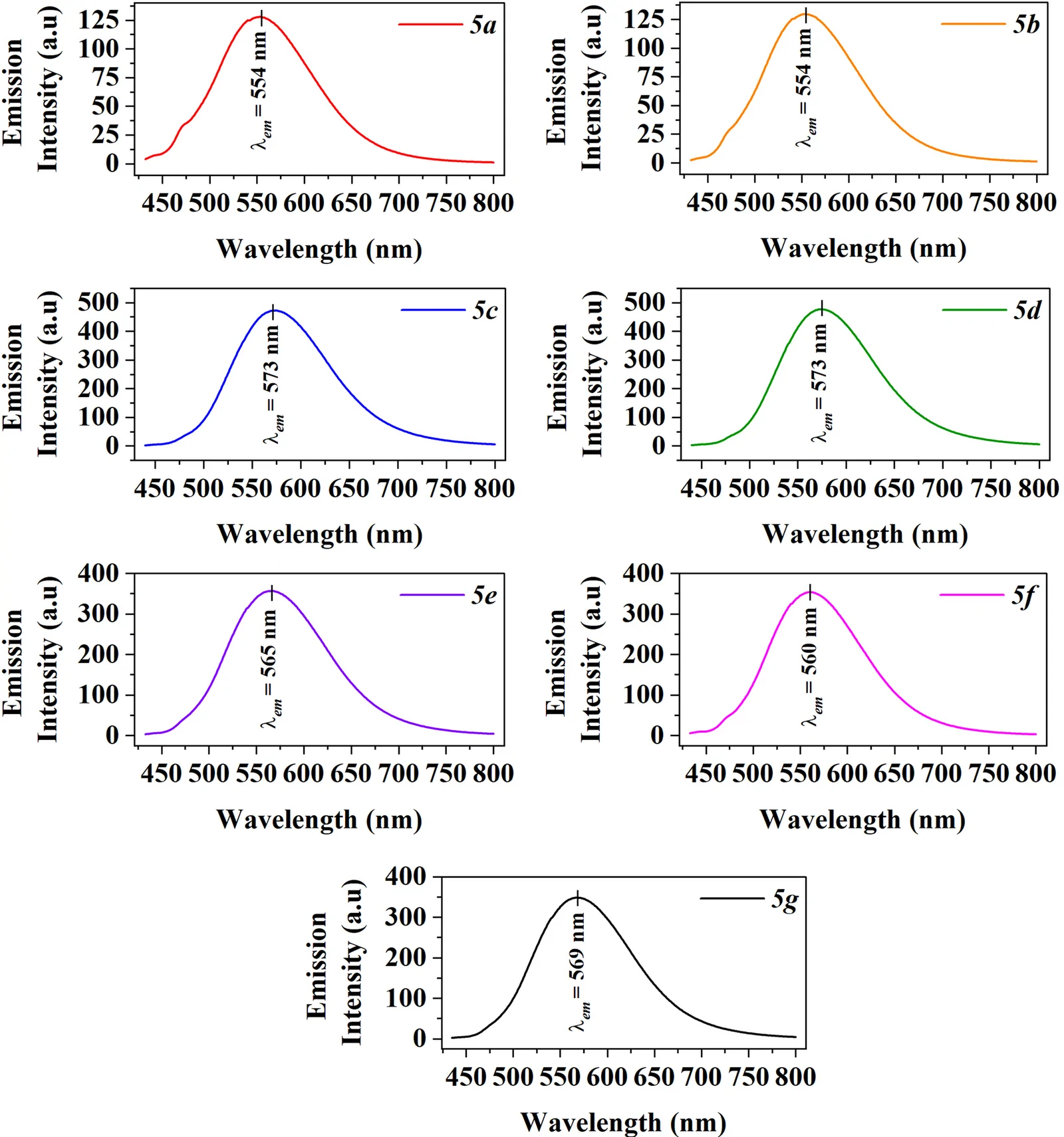
Photoluminescence emission spectra of compounds 5a–g in DMF (1 × 10^− 4^ M)

**Fig. 4:**
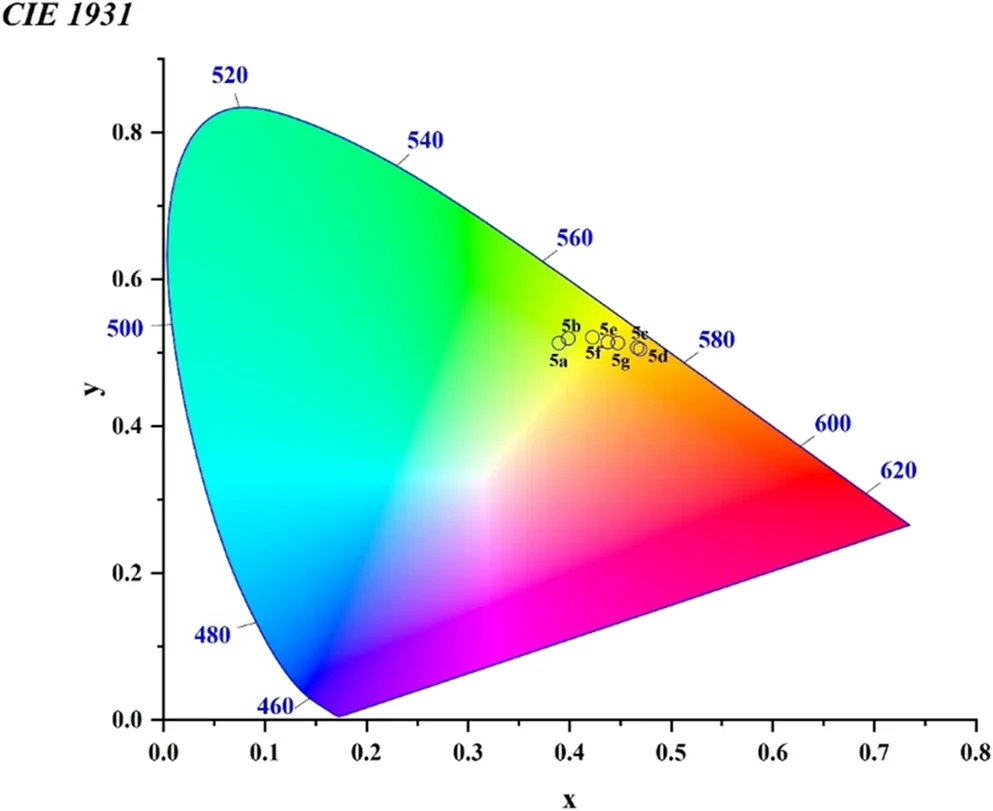
CIE 1931 chromaticity diagram showing the emission color coordinates of compounds 5a–g

The photoluminescence (PL) emission spectra for compounds 5a–g were obtained in DMF at a concentration of 1 × 10^− 4^ M, at room temperature, with excitation at their respective absorption maxima (Fig. 3). For all seven compounds, the emission maxima (λ_em_) occurred within the yellow-green portion of the visible spectrum, in the range 554–575 nm. An intramolecular charge-transfer (ICT) process within the hydrazone-dihydrothiazole π-conjugated framework is proposed as a plausible contributor to the excited-state behavior of these compounds, consistent with the broad, featureless emission profile observed for all seven derivatives [36]. Further support for this was derived from the CIE 1931 chromaticity coordinates of all the compounds (Fig. 4), which clustered within the yellow-green region, with compounds 5a and 5b positioned at lower x values (~ 0.37) and compounds 5c and 5d at higher x values (~ 0.47), consistent with their red-shifted emission maxima.

The nature of the substituent at the C-4 position of the dihydrothiazole ring was found to play an important role in tuning the emission maxima, following trends similar to those observed in the UV-Vis absorption spectra. Compounds 5c and 5d, bearing the electron-donating 3,4-dimethoxyphenyl group, showed the largest bathochromic shift (λ_em_ = 575 nm) along with the highest emission intensity (~ 490 a.u). This trend is consistent with reports in which electron-donating substituents narrow the LUMO–HOMO energy gap and promote a bathochromic shift in emission, suggesting that the dimethoxy groups in 5c and 5d may similarly narrow the S_1_–S_0_ energy gap through greater excited-state charge-transfer character [37, 38]. In contrast, 5a and 5b, bearing the electron-withdrawing 3-nitrophenyl group, showed the most blue-shifted emission maxima (λ_em_ = 554 nm) and the lowest emission intensity (~ 130 a.u). The lower emission intensity observed for 5a and 5b aligns with reports on nitro-substituted fluorophores, which are known to favor non-radiative decay pathways such as photoinduced electron transfer (PET), possibly contributing to the observed quenching in the present system [39, 40]. Compounds 5e and 5f, each bearing a 3-chlorophenyl substituent at the C-4 position, demonstrated intermediate emission maxima at 565 nm and 560 nm, respectively, with a moderate emission intensity of ~ 360 a.u, consistent with the weak electron-withdrawing effect of the chlorine substituent. Although both compounds were expected to exhibit identical emission maxima according to the UV-Vis absorption data, the additional blue shift observed for 5f relative to 5e may be attributed to the bromine atom on the pyridine ring, whose combined presence with the chlorine atom at the C-4 position may further enhance spin-orbit coupling and promote non-radiative intersystem crossing to the triplet state - a heavy-atom effect previously reported in related systems [41, 42]. Compound 5 g, bearing a 3-methoxyphenyl substituent, displayed an emission maximum of 569 nm and a moderate emission intensity of ~ 310 a.u. This result supports that the increasing bathochromic shift of the emission maxima correlates with the electron-donating strength of the substituent. The shift is smaller than that observed with the 3,4-dimethoxy substituent, since only one methoxy group is present in this case. In summary, the emission maxima follow the order: 5c and 5d (575 nm) > 5 g (569 nm) > 5e (565 nm) > 5f (560 nm) > 5a and 5b (554 nm).

#### Stokes Shift Analysis

The absorption and emission maxima, the corresponding Stokes shifts, and the quantum efficiencies of compounds 5a–g are summarized in Table 1. The Stokes shifts across the series range from 142 to 155 nm, calculated using the formulae Stokes shifts(∆λ)=λ_emission_ - λ_absorption_. Among the series, compounds 5a and 5b exhibit the minimal Stokes shift of 142 nm, whereas 5c and 5d display the maximal value at 155 nm, and the rest (5e–5 g) lie in an intermediate range from 147 to 154 nm. As seen across the series of compounds, the narrow range of Stokes shifts indicates that the pathway for excited-state relaxation remains largely consistent among all seven compounds, regardless of the variation in the electronic properties of their C-4 aryl substituents. The quantum efficiency of the synthesized compound was measured in solution by the absolute method. The quantum efficiency of thiazoles 5a-g varies between 1.64% (5c) to 5.54% (5 g). This shows that the efficiency of radiative and non-radiative deactivation of these molecules differs. Among thiazoles, 5 g exhibited the highest quantum efficiency (5.54%), indicating that excitation-state relaxation occurred predominantly via fluorescence. However, 5c had the lowest ΦF value (1.64%), indicating enhanced non-radiative deactivation. The remaining derivatives exhibited medium quantum efficiencies, in decreasing order: 5 g > 5a > 5b > 5e > 5f > 5d > 5c. The observed trends are due to structural differences, the presence of different functional groups in the molecule, and molecular rigidity or intramolecular charge transfer.

**Table 1:** Absorption and emission maxima with corresponding stokes shifts of compounds 5a–g

Compounds	λ_max_ (nm)	λ_em_ (nm)	Stokes Shift (∆λ, nm)	Quantum Efficiency (%)
5a	412	554	142	4.65
5b	412	554	142	4.04
5c	420	575	155	1.64
5d	420	575	155	2.55
5e	413	565	152	3.92
5f	413	560	147	3.36
5 g	415	569	154	5.54

## Conclusion

In summary, this paper reports a straightforward one-pot synthesis method for the preparation of seven novel dihydrothiazole-hydrazone compounds. FTIR, 1 H NMR, 13 C NMR, and ESI-MS analysis characterized them. We have demonstrated that the absorption occurred in the 412–420 nm range and emission occurred in the yellow-green region spanning 554–575 nm, which is closely tied to the electronic character of the aryl substituent at the C-4 position of the dihydrothiazole ring. Electron-donating groups produced a bathochromic shift and increased emission. In contrast, electron-withdrawing groups resulted in decreased emission intensity, a pattern also observed in the Stokes shifts recorded across the series (142–155 nm). Such a structure-based adjustable framework that allows substituent tuning without requiring structurally distinct pathways for each compound is ideal for exploring its applications in metal-ion sensing, biological imaging, and optoelectronic devices. 

## Supplementary Information

Below is the link to the electronic supplementary material.

Supplementary Material 1 (DOCX 5.60 MB)

## Data Availability

Data will be made available on request.
