# Supplementary material for: One-Pot Synthesis of Dihydrothiazole-Hydrazone Hybrids and Evaluation of Their Absorption and Emission Properties
Source: J Fluoresc. 2026 Sep 29;36(11):6043–53. doi: 10.1007/s10895-026-04955-6 (PMC13722728; doi:10.1007/s10895-026-04955-6)
Supplement: Supplementary file 1 [file 10895_2026_4955_MOESM1_ESM.docx]

**One-Pot Synthesis of Dihydrothiazole-Hydrazone Hybrids and Evaluation of their Absorption and Emission Properties**

Charan D.C., Yogeesh M., Nitinkumar S. Shetty*

Manipal Institute of Technology, Manipal Academy of Higher Education, Manipal, India

Corresponding author: *nitin.shetty@manipal.edu

**2. Experimental**

**2.3 Spectral Characterization**

**2.3.1 Synthesis of 2-((*Z*)-((3-chloropyridin-4-yl)methylene)hydrazono)-4-(3-nitrophenyl)-3-phenyl-2,3-dihydrothiazole *(5a)***

**Fig. S1** FTIR spectrum ***(5a)***

**Fig. S2** ^1^H NMR spectrum of ***(5a)***

**Fig. S3** ^13^C NMR spectrum of ***(5a)***

**Fig. S4** ESI-MS spectrum of ***(5a)***

**2.3.2 Synthesis of 2-((*Z*)-((3-bromopyridin-4-yl)methylene)hydrazono)-4-(3-nitrophenyl)-3-phenyl-2,3-dihydrothiazole *(5b)***

**Fig. S5** FTIR spectrum ***(5b)***

**Fig. S6** ^1^H NMR spectrum of ***(5b)***

**Fig. S7** ^13^C NMR spectrum of ***(5b)***

**Fig. S8** ESI-MS spectrum of ***(5b)***

**2.3.3 Synthesis of 2-((*Z*)-((3-chloropyridin-4-yl)methylene)hydrazono)-4-(3,4-dimethoxyphenyl)-3-phenyl-2,3-dihydrothiazole *(5c)***

**Fig. S9** FTIR spectrum ***(5c)***

**Fig. S10** ^1^H NMR spectrum of ***(5c)***

**Fig. S11** ^13^C NMR spectrum of ***(5c)***

**Fig. S12** ESI-MS spectrum of ***(5c)***

**2.3.4 Synthesis of 2-((*Z*)-((3-bromopyridin-4-yl)methylene)hydrazono)-4-(3,4-dimethoxyphenyl)-3-phenyl-2,3-dihydrothiazole *(5d)***

**Fig. S13** FTIR spectrum ***(5d)***

**Fig. S14** ^1^H NMR spectrum of ***(5d)***

**Fig. S15** ^13^C NMR spectrum of ***(5d)***

**Fig. S16** ESI-MS spectrum of ***(5d)***

**2.3.5 Synthesis of 4-(3-chlorophenyl)-2-((*Z*)-((3-chloropyridin-4-yl)methylene)hydrazono)-3-phenyl-2,3-dihydrothiazole *(5e)***

**Fig. S17** FTIR spectrum ***(5e)***

**Fig. S18** ^1^H NMR spectrum of ***(5e)***

**Fig. S19** ^13^C NMR spectrum of ***(5e)***

**Fig. S20** ESI-MS spectrum of ***(5e)***

**2.3.6 Synthesis of 2-((*Z*)-((3-bromopyridin-4-yl)methylene)hydrazono)-4-(3-chlorophenyl)-3-phenyl-2,3-dihydrothiazole *(5f)***

**Fig. S21** FTIR spectrum ***(5f)***

**Fig. S22** ^1^H NMR spectrum of ***(5f)***

**Fig. S23** ^13^C NMR spectrum of ***(5f)***

**Fig. S24** ESI-MS spectrum of ***(5f)***

**2.3.7 Synthesis of 2-((*Z*)-((3-chloropyridin-4-yl)methylene)hydrazono)-4-(3-methoxyphenyl)-3-phenyl-2,3-dihydrothiazole *(5g)***

**Fig. S25** FTIR spectrum ***(5g)***

**Fig. S26** ^1^H NMR spectrum of ***(5g)***

**Fig. S27** ^13^C NMR spectrum of ***(5g)***

**Fig. S28** ESI-MS spectrum of ***(5g)***
